# CRISPR-Cas9 identifies growth-related subtypes of glioblastoma with therapeutical significance through cell line knockdown

**DOI:** 10.1186/s12885-023-11131-7

**Published:** 2023-08-14

**Authors:** Nannan Zhao, Siyuan Weng, Zaoqu Liu, Hui Xu, Yuqin Ren, Chunguang Guo, Long Liu, Zhenyu Zhang, Yuchen Ji, Xinwei Han

**Affiliations:** 1 Department of Interventional Radiology, The First Affiliated Hospital of Zhengzhou University, Zhengzhou, Henan 450052 China; 2 Department of Neurosurgery, The First Affiliated Hospital of Zhengzhou University, Zhengzhou, Henan 450052 China; 3 Department of Respiratory and Critical Care Medicine, The First Affiliated Hospital of Zhengzhou University, Zhengzhou, China; 4 Department of Endovascular Surgery, The First Affiliated Hospital of Zhengzhou University, Zhengzhou, China; 5 Department of Hepatobiliary and Pancreatic Surgery, The First Affiliated Hospital of Zhengzhou University, Zhengzhou, China

**Keywords:** CRISPR-Cas9, Glioblastoma, Tumor heterogeneity, Copy number variants, Precision medicine

## Abstract

**Background:**

Glioblastoma (GBM) is a type of highly malignant brain tumor that is known for its significant intratumoral heterogeneity, meaning that there can be a high degree of variability within the tumor tissue. Despite the identification of several subtypes of GBM in recent years, there remains to explore a classification based on genes related to proliferation and growth.

**Methods:**

The growth-related genes of GBM were identified by CRISPR-Cas9 and univariate Cox regression analysis. The expression of these genes in the Cancer Genome Atlas cohort (TCGA) was used to construct growth-related genes subtypes (GGSs) via consensus clustering. Validation of this subtyping was performed using the nearest template prediction (NTP) algorithm in two independent Gene Expression Omnibus (GEO) cohorts and the ZZ cohort. Additionally, copy number variations, biological functions, and potential drugs were analyzed for each of the different subtypes separately.

**Results:**

Our research established multicenter-validated GGSs. GGS1 exhibits the poorest prognosis, with the highest frequency of chr 7 gain & chr 10 loss, and the lowest frequency of chr 19 & 20 co-gain. Additionally, GGS1 displays the highest expression of EGFR. Furthermore, it is significantly enriched in metabolic, stemness, proliferation, and signaling pathways. Besides we showed that Foretinib may be a potential therapeutic agent for GGS1, the worst prognostic subtype, through data screening and in vitro experiments. GGS2 has a moderate prognosis, with a slightly higher proportion of chr 7 gain & chr 10 loss, and the highest proportion of chr 19 & 20 co-gain. The prognosis of GGS3 is the best, with the least chr 7 gain & 10 loss and EGFR expression.

**Conclusions:**

These results enhance our understanding of the heterogeneity of GBM and offer insights for stratified management and precise treatment of GBM patients.

**Supplementary Information:**

The online version contains supplementary material available at 10.1186/s12885-023-11131-7.

## Introduction

Glioblastoma (GBM) is an adult malignant tumor with a median survival of around 16 months [1]. GBM exhibits extensive intratumoral heterogeneity, representing a major barrier to effective therapy [2]. According to previous studies, patients with MGMT methylation showed greater benefit from chemoradiation [3]. Therefore, the reclassification of GBM and adoption of precision medicine strategies with stratified management and personalized treatment can significantly improve efficacy. Verhaak et al. proposed that based on transcriptome data GBM can be divided into four types: Proneural, Neural, Classical, and Mesenchymal [4]. This classification has had a profound impact on the research of GBM, but this classification does not take into account the role of specific genes, particularly growth-related genes.

CRISPR-Cas9 technology is a stable, efficient, and simple gene editing tool that allows for the targeted editing of specific genes [5]. Since its inception, this technology has been widely utilized in various biomedical fields, offering new avenues for studying the mechanisms underlying tumor development and progression, as well as promising new strategies for treating tumors [6, 7]. Based on CRISPR-Cas9 technology, the Depmap database was established by Tsherniak et al. They used the technology to specifically knockout genes in a variety of cancer cell lines, combined with the growth of cancer cell lines after the knockout, and then predicted genes necessary for cancer growth [8]. Chen et al. screened osteosarcoma growth-related genes using the DepMap and constructed a prognostic risk model. To further verify that LARS is an essential gene for osteosarcoma growth, cellular experiments were conducted. It provides a new perspective on the risk stratification of osteosarcoma [9]. Similarly, Ho et al. used the lung adenocarcinoma growth-related genes obtained from the DepMap database to perform consensus clustering, which divided patients into subgroups with different prognoses, further enriching the classification of lung adenocarcinoma [10].

We utilized the DedMap database and the Cancer Genome Atlas (TCGA) transcriptome data to reclassify GBM patients, which was validated in both the GEO cohorts and the ZZ cohort. We analyzed the differences in biological functions, such as copy number variations, among the three subtypes. Specifically, we delved deeper into the possible biological mechanisms responsible for the worst prognosis in the GGS1 subtype and predicted potential drugs that could target it. By stratifying patients based on growth-related genes, our findings offer valuable insights and ideas for further research and precision medicine in GBM.

## Materials and methods

### Data sources and processing

**GBM RNA-seq data.** From the TCGA, we obtained clinical information and RNA-seq data of 143 samples that were pathologically diagnosed as GBM, which we downloaded. Next, the data were further processed to convert the raw counts in Fragments Per Kilobase Million (FPKM) normalization to Transcripts Per Million (TPM) values and further transformed into log2 (TPM + 1) format. Table S1 contains information on samples that have been pathologically diagnosed with GBM in this cohort.

**GBM microarray data.** We collected two independent cohorts, GSE108474 and GSE7696, from the Gene Expression Omnibus (GEO). Samples with pathological diagnoses of GBM were selected. Information on samples pathologically diagnosed with GBM in this two cohorts is provided in Table S1. The Affymetrix GPL570 platform (Human Genome U133 Plus 2.0 Array) was used to retrieve normalized matrix files for the GEO cohorts. The Affy package implemented the robust multi-array averaging (RMA) algorithm to process raw data from Affymetrix.

**In-house data.** The Human Scientific Ethics Committee of the First Affiliated Hospital of Zhengzhou University approved this study (No. 2019-KY-176) and complied with the Declaration of Helsinki. Obtain informed consent from all patients who provide tumor specimens before participating in the research. The pertinent clinical information for this cohort is presented in Table S1. As described in a previous study [11], RNA samples assessed by quantification and identification will undergo subsequent library preparation, generated using the NEBNext Ultratmrnlibrary Prep Kit for Illumina (NEB, USA), and sequenced on the Illumina hiseq platform. High-quality clean data (clean reads) obtained after quality control of raw data for downstream analysis.

### Growth-related genes were found in the DepMap database based on the CERES score

The Dependency Map (DepMap, https://depmap.org/portal/) portal, established using RNAi and CRISPR-Cas9 technology, is a database of thousands of cell lines from dozens of tumors [8]. The importance of genes for cell proliferation and growth is determined by their effect on cell growth when knocked down. However, cancer cells often have higher genomic copy number variations (CNVs), leading to DNA double-strand breaks and damage responses when the single-guide RNA (SgRNA)-Cas9 complex targets these regions. This can inhibit cell growth and cause false positives in gene knockout studies [12]. To address these issues, the CERES score was developed as a new parameter for evaluating gene necessity. This unbiased method estimates gene dependence while considering copy number effects at all levels of CNVs. The lower the CERES score, the more necessary the gene is for cell growth. This indicates that genes with lower CERES scores are more critical for cell growth [13].

As previously reported [9], we performed the genome-scale CRISPR-Cas9 screening in GBM to find growth-related genes with prognostic significance. The screening process involved the following steps: (1) The CERES algorithm was applied to calculate the dependency scores of 17,386 candidate genes. (2) Genes with CERES scores < -1 (n = 699) in more than 80% of GBM cell lines (n = 61) were identified as growth-related genes (Table S2). (3) The screening of growth-related genes with prognostic significance was conducted through univariate analysis in the TCGA cohort (n = 143).

### Subtypes were established based on growth-related genes

Univariate Cox analysis was conducted on the TCGA cohort, selecting 12 growth-related genes with prognostic significance (Table S3). Then, the expression differences of these 12 genes in both adjacent and cancerous tissues were analyzed in the TCGA cohort. The expression levels of these genes were used for unsupervised consensus clustering, which was performed using the Partitioning Around Medoids (PAM) algorithm [14]. To ensure robust clustering, 500 iterations were performed, with each iteration resampling 80% of patients in the TCGA cohort [15]. The maximum number of clusters was set to 9, and the consensus cumulative distribution function and consensus heatmap were used to evaluate K values. Principal component analysis (PCA) is one of the most widely used dimensionality reduction algorithms, based on which we explore the similarity of samples from the same subtype at the transcriptome level.

### Identify feature genes for subtype validation

We performed a within-group difference analysis for the three groups via the limma R package. The order of genes was sorted decreasingly by logFC. We selected the top 500 genes of these three groups as feature genes for GGS1, GGS2, and GGS3, respectively (Table S4).

### Subtype validation based on the NTP algorithm

The nearest template prediction (NTP) is an algorithm that can be flexibly applied to multi-class prediction in across-platform and across-species, without any parameter optimization [16]. This method can predict the corresponding subtypes by signature gene list and testing datasets while giving prediction confidence [17]. In this study, we evaluated the robustness of this classification by running the NTP algorithm based on GGSs signature genes via the CMScaller package in three cohorts: GSE7696, GSE108474, and ZZ cohort. Additionally, we employed the NTP method to classify the cell lines corresponding to different subtypes. To ensure the accuracy of classification, samples with FDR > 0.05 were eliminated.

### Copy number variations analysis of GGSs

We obtained data on copy number variations from the UCSC Xena website (https://xena.ucsc.edu/). The maftools R package was used to analyze the top 15 segments with the highest frequency of copy number variations. To fully understand the overall copy number variations between different subtypes, as mentioned earlier [18, 19], we used the GISTIC2 algorithm to calculate the GISTIC score and variation frequency.

### Analysis of potential biological functions

We conducted the functional analysis using gene set enrichment analysis (GSEA) through the clusterProfiler R package [20]. This approach is distinct from traditional enrichment analysis methods in that it considers the global trend of gene expression. The order of genes of each subtype was sorted decreasingly by logFC to serve as input files, and pathways with larger NES values were selected for visual display. To increase the persuasion of our findings, we scored each sample’s pathway by another gene set enrichment algorithm, the gene-set variation analysis (GSVA) [21]. Differential analysis was carried out using the limma R package based on these pathway scores for the three subtypes. We identified pathways that displayed statistically significant differential expression, as determined by an FDR threshold of less than 0.05 and a log fold change greater than 0, and selected them for visualization. To verify our previous functional analysis, we also obtained protein expression data from TCPA (The Cancer Proteome Atlas, https://www.tcpaportal.org/tcpa/).

### Exploration of potential therapeutic agents for GGS1

As previously reported [22], our study aimed to identify potential therapeutic agents for GGS1, the subtype with the worst prognosis. We utilized two databases, the cancer therapeutics response portal (CTRP) and profiling of relative inhibition simultaneously in mixtures (PRISM), to perform the following analyses: (1) Ridge regression was performed using the pRRophetic package to predict the AUC value of each sample to the drug, and the lower the AUC value means the more sensitive to the drug response; (2) Wilcox test analysis was performed to determine drugs with significant differences. The differences between GGS1 and GGS2, GGS1 and GGS3 were analyzed in PRISM and CTRP databases, respectively, and drugs with P values less than 0.05 were defined as GGS1 versus the other two types of differentially sensitive drugs. (3) AUC mean values were calculated for each subtype under different drugs to select drugs that were more sensitive to GGS1. In the PRISM database and CTRP database, drugs with AUC mean values smaller than the other two types in GGS1 were screened. (4) The drugs obtained in the second and third steps were intersected to be potential therapeutic drugs for GGS1.

### Reagents and cell line

Foretinib (Lot: 114,907) was procured from TargetMol. CCK8 (Lot: 211104Z01-05) was obtained from US EVERBRIGHT. The human GBM cell line U251 was gifted by Professor Zhenyu Zhang, Department of Neurosurgery, the First Affiliated Hospital of Zhengzhou University. The cells were cultured in DMEM (high glucose) supplemented with 10% FBS at 37 °C in a 5% CO2 environment.

### CCK8 assay

CCK8 assays were performed according to the manufacturer’s guidelines. Briefly, we seeded 5,000 cells in 100 µL of culture medium per well in a 96-well plate. Following a 5-hour period for cell attachment, varying concentrations of Foretinib (0.5µM, 1µM, 2µM, 5µM, 10µM, 25µM, 50µM) were added to the wells. The plate was then placed in a controlled incubator and incubated for the specified durations of 24 and 48 h. After the incubation, 10µL of CCK-8 solution was added to each well, and the plate was further incubated for 2 h. Finally, the absorbance at 450 nm was measured using a microplate reader. The half-maximal inhibitory concentration (IC50) was determined using GraphPad Prism 9.0 software (GraphPad; San Diego, CA, USA).

### Statistical analysis

The data was processed, analyzed, and visualized using R version 4.1.0. and GraphPad Prism (GraphPad Software 9.0). Continuous variables were analyzed using both the Wilcoxon rank-sum test and T-test, while categorical variables were compared using the Chi-square test. Survival curves were generated using the survival package and analyzed using the log-rank test. The hazard ratio (HR) of the variables was calculated using univariate Cox regression analysis, and independent prognostic factors were identified using multiple Cox regression analysis. All statistical tests were considered two-sided, and a P-value less than 0.05 indicated statistical significance.

## Results

### Identify growth-related genes of GBM by CRISPR-Cas9 technology

**Fig. 1:**
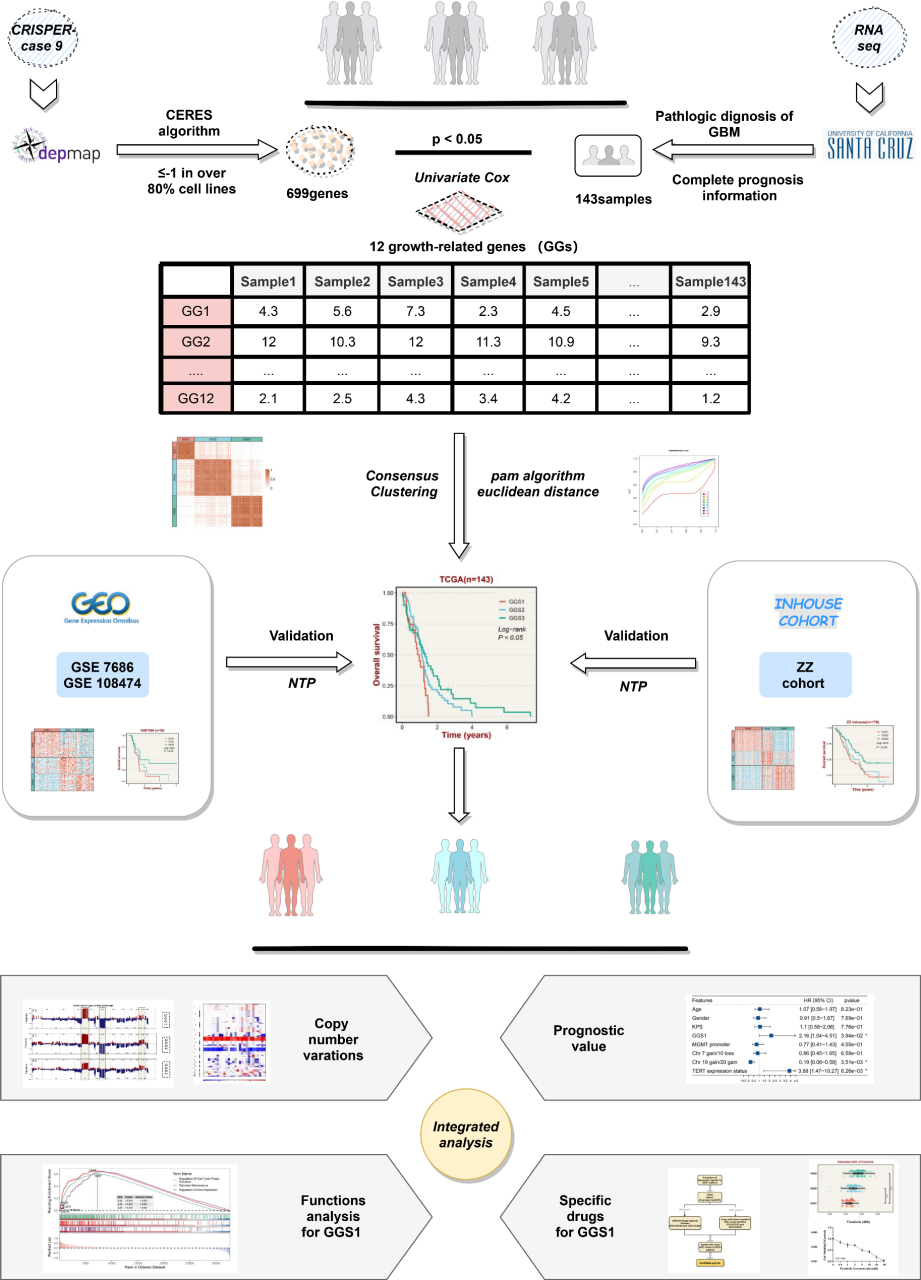
Analysis flow chart of this study

**Fig. 2:**
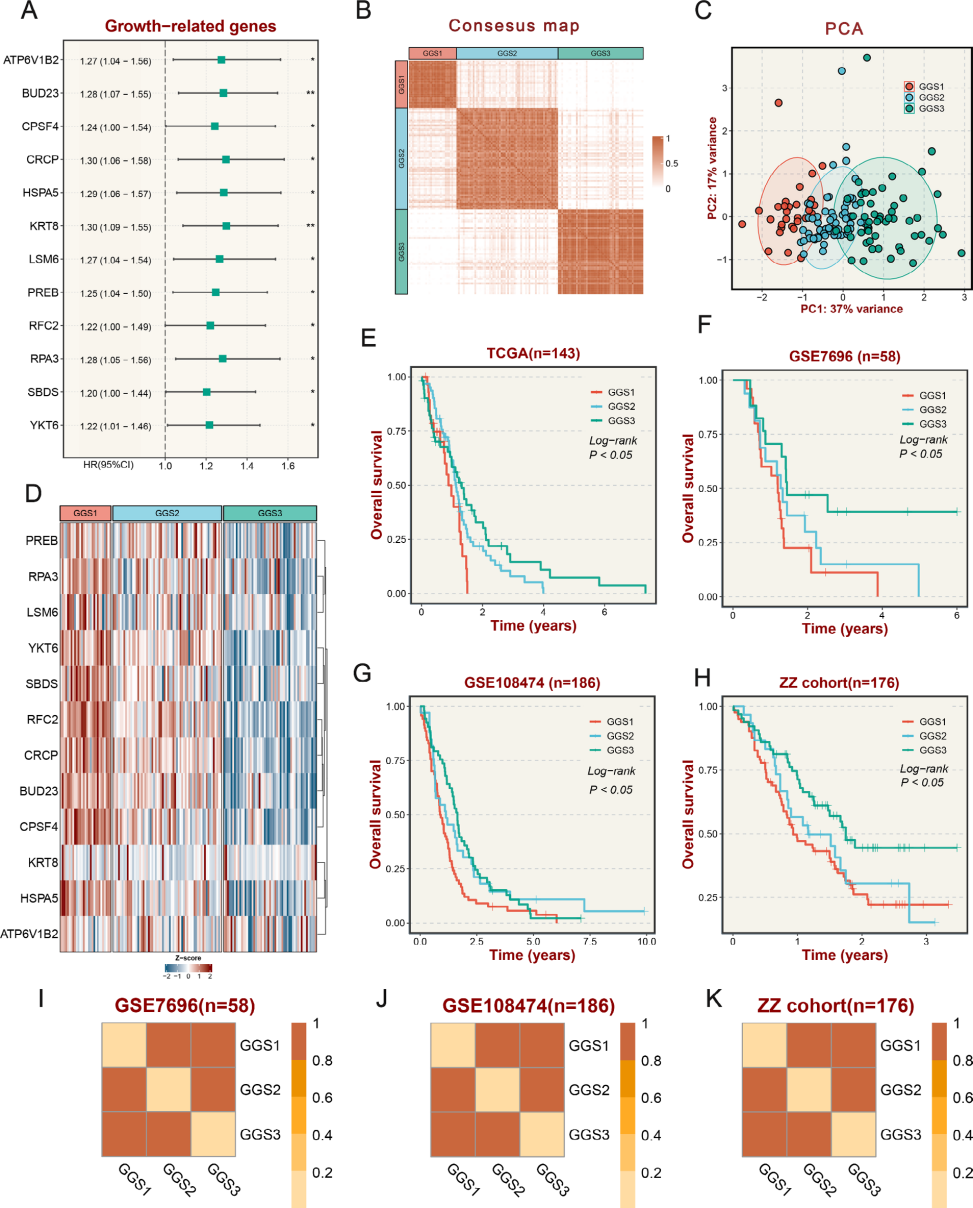
**The establishment and validation of growth-related genes subtypes (GGSs). (A)** Univariate analysis identified 12 growth-related genes. **(B)** Consensus map of clustering in the TCGA cohort. **(C)** PCA analysis based on growth-related genes. **(D)** The expression of growth-related genes in three subtypes. **(E)** KM survival curve analysis of GGSs in the TCGA cohort using a log-rank test. **(F)** KM survival curve analysis of GGSs in the GSE108474 cohort using a log-rank test. **(G)** KM survival curve analysis of GGSs in the GSE7696 cohort using a log-rank test. **(H)** KM survival curve analysis of GGSs in the ZZ cohort using a log-rank test. **(I-K)** SubMap plots, located in the bottom panel, evaluated expressive similarity between corresponding subtypes from two different cohorts

Flowchart depicting the entirety of this study (Fig. 1). To screen genes essential for GBM growth, we performed genome-wide screening using DepMap, which revealed 61 GBM cell lines with 17,386 genes. We further screened 699 genes with CERES scores < -1 in over 80% of GBM cell lines. Based on the expression of these 699 genes in the TCGA cohort, a univariate COX analysis was conducted, which resulted in the selection of twelve growth-related genes (Table S3) that had significant prognostic value. Notably, The HR values of these genes were greater than 1, indicating that they were associated with a poor prognosis (Fig. 2A). Besides, in contrast to adjacent non-cancerous samples within the TCGA cohort, we observed that these 12 growth-related genes were predominantly expressed at high levels in cancer tissues (Figure S1).

### Subtype classification based on growth-related genes

The expression of growth-related genes in the TCGA cohort was used to construct a matrix. The consensus clustering was performed via the ConsensusClusterPlus package [23] based on this matrix, and the samples were initially divided into 2–9 clusters (k = 2–9). According to the comprehensive analysis of cumulative distribution function (CDF) curves and consensus clustering matrix heatmap (Fig. 2B, S2A), we selected the optimal k value of 3. According to the optimal k value, all samples were divided into three growth-related genes subtypes (GGSs). Principal Component Analysis (PCA) indicated that significant differences in subtypes were validated at the growth gene level (Fig. 2C). Meanwhile, the expression of growth-related genes was highest in GGS1, followed by GGS2, and lowest in GGS3 (Fig. 2D). As expected, GGS1 had the worst prognosis, GGS3 had the best prognosis, and GGS2 was intermediate (Fig. 2E).

### Validation in microarray cohort and in-house RNAseq cohort

We performed validation in two microarray cohorts (platform GPL570) and one in-house cohort. Using the expression of feature genes (Table S3), we utilized the NTP algorithm to predict the subtypes of the validation sets. To ensure the accuracy of the results, samples with FDR greater than 0.05 were filtered out. Each validated dataset is divided into three subtypes, GGS1-3. The Kaplan-Meier prognostic analysis was the same result as the TCGA cohort (Fig. 2F-H). According to the classification results, the feature genes had similar expressions in the corresponding subtypes of the validation sets (Figure S2B-D). Besides, the submap analysis also showed a high similarity of common signature genes between the TCGA cohort and the validation cohort (Fig. 2I-K).

### The differences in copy number variations and frequency among GGSs

**Fig. 3:**
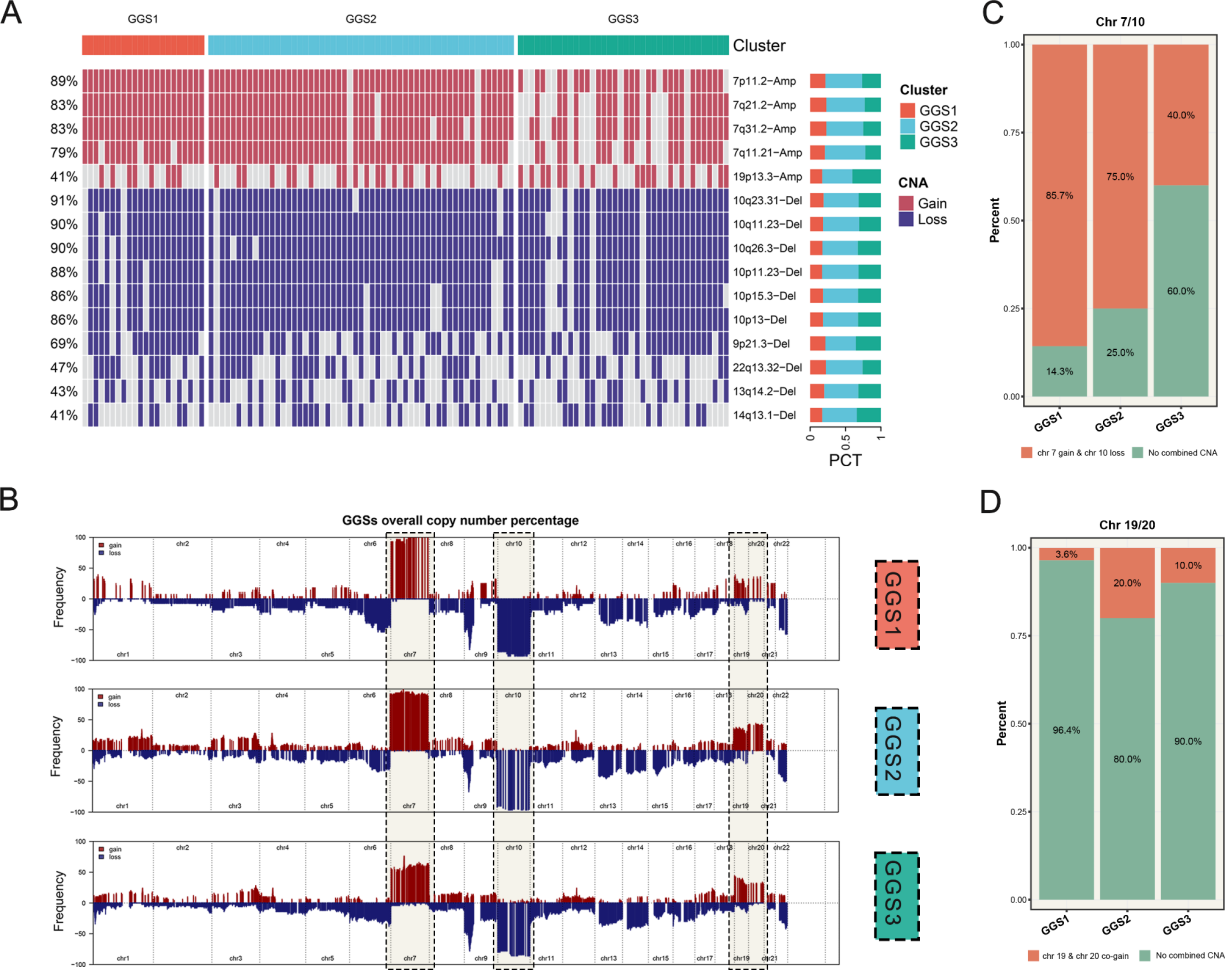
**The differences in copy number variations and frequency between GGSs. (A)** The distribution of the first 5 segments of chromosome gain and the first 10 segments of chromosome loss between GGS1-3. **(B)** The copy number variations frequency landscape of GGS1-3. **(C)** The distribution of chr 7 gain & chr 10 loss in GGS1-3. **(D)** The distribution of chr 19 & 20 co-gain in GGS1-3

We observed that among the top 15 segments with the highest frequency of copy number variations, amplified segments were predominantly located at chr 7 and deleted segments were predominantly located at chr 10 (Fig. 3A). To explore the overall picture of copy number variations, we calculated the GISTIC score and copy number alteration frequency for each subtype using the GISTIC2 algorithm [18]. We found that the frequency of alterations in chr 7 amplification was highest in GGS1, slightly lower in GGS2, and lowest in GGS3 (Fig. 3B). The frequency of chr 10 loss was lowest in GGS3(Fig. 3B). These findings were consistent with the trend observed in the row copy number, and we also found GGS1 has a higher frequency of chr 7 gain & chr 10 loss than GGS2 (Figure S3A-C). In addition, the frequency of chr 19 & 20 co-gain was highest in GGS2, followed by GGS3, and lowest in GGS1 (Fig. 3B). To further analyze the distribution of samples in the three subtypes. After referring to previous studies [24], we discovered that the proportion of samples with chr 7 gain and chr 10 loss was highest in GGS1, followed by GGS2, while GGS3 had the lowest proportion (Fig. 3C). Chr 19 & 20 co-gain exhibits the highest proportion of samples at GGS2, followed by GGS3, with GGS1 exhibiting the least (Fig. 3D).

### The underlying biological explanation for the poor prognosis of GGS1

**Fig. 4:**
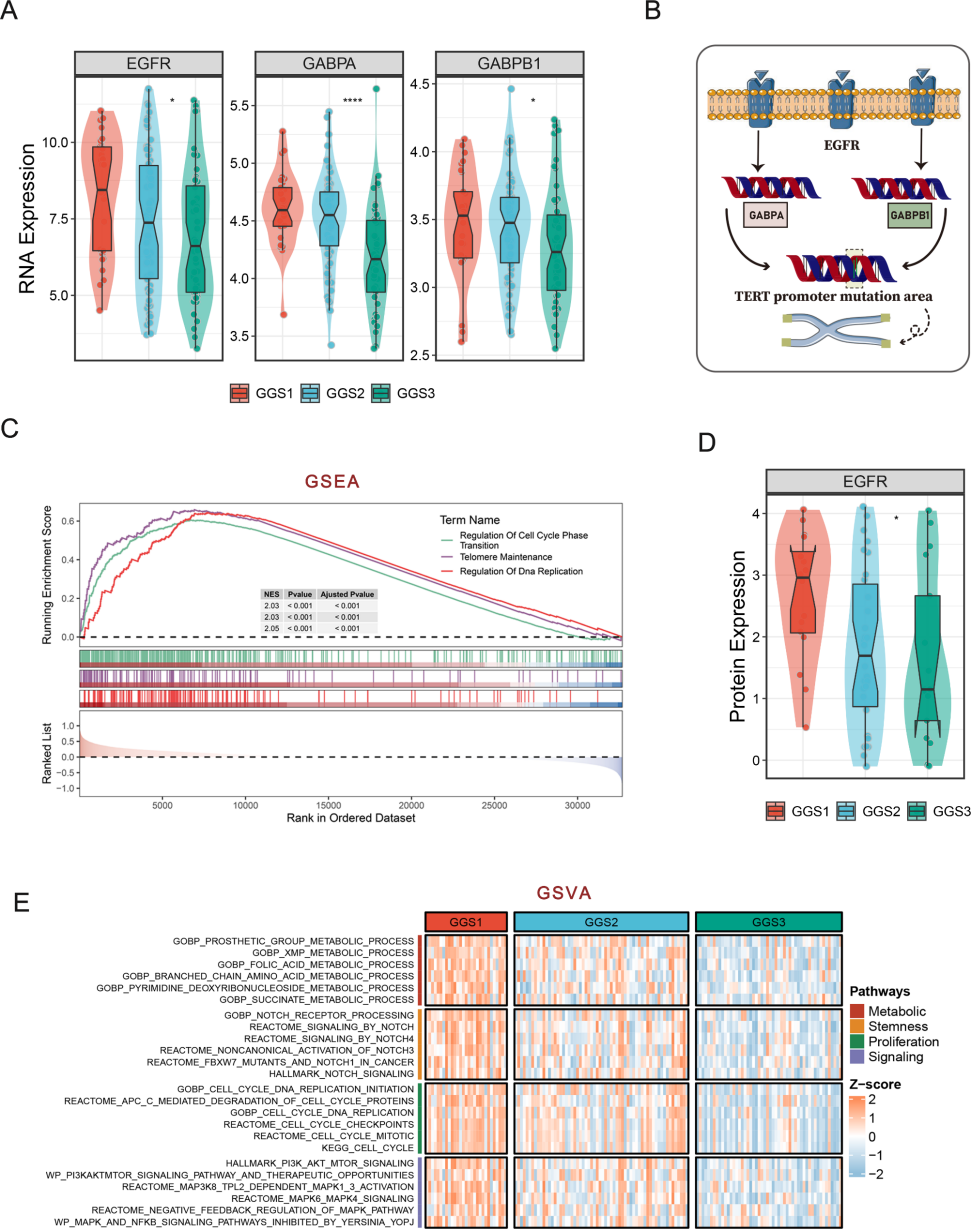
**The underlying biological explanation for the poor prognosis of GGS1. (A)** The distribution of EGFR, GABPA, GABPB1 transcriptome expression in GGS1-3. **(B)** Mechanistic mapping of EGFR acting on the TERT promoter mutated region via GABPA and GABPB1 couplets. **(C)** GSEA enrichment analysis results for GGS1. **(D)** The distribution of EGFR protein expression among GGS1-3. **(E)** GSVA enrichment analysis results for GGS1-3

Our analysis focused on investigating the biological functions of GGS1, which is associated with the worst prognosis. Referring to previous findings [24], we observed that TERT expression status accounted for a relatively high proportion of cases in GGS1 (Figure S3D). At the same time, TERT expression status is closely related to TERT promoter status, and TERT expression status with TERT promoter mutation is mostly present [24]. As expected, the proportion of TERT promoter mutation in GGS1 was the highest (Figure S3E). However, due to insufficient information on TERT promoter mutation in the TCGA cohort, we validated it in the in-house cohort (Figure S3F). In addition, we found that both EGFR and GABP were highly expressed in GGS1 (Fig. 4A), and EGFR could promote cell proliferation and immortality by GABP acting on TERT-promoter mutated regions (Fig. 4B) by previous findings [25]. Next, we performed GSEA enrichment analysis, which revealed that pathways promoting proliferation and immortality were enriched in GGS1, including regulation of cell cycle phase transition, telomere maintenance, and regulation of DNA replication (Fig. 4C). Besides, the protein expression level of EGFR was highly expressed in GGS1 (Fig. 4D). To further compare differences in pathway activity between subtypes, GSVA enrichment analysis was performed. Enriched pathways are divided into four categories, metabolism, stemness, proliferation, and signaling. We discovered that GGS1 is activated and highly expressed in all four classes of functions, whereas GGS2 and GGS3 are not (Fig. 4E).

### Comparison with recognized characteristics of GBM

**Fig. 5:**
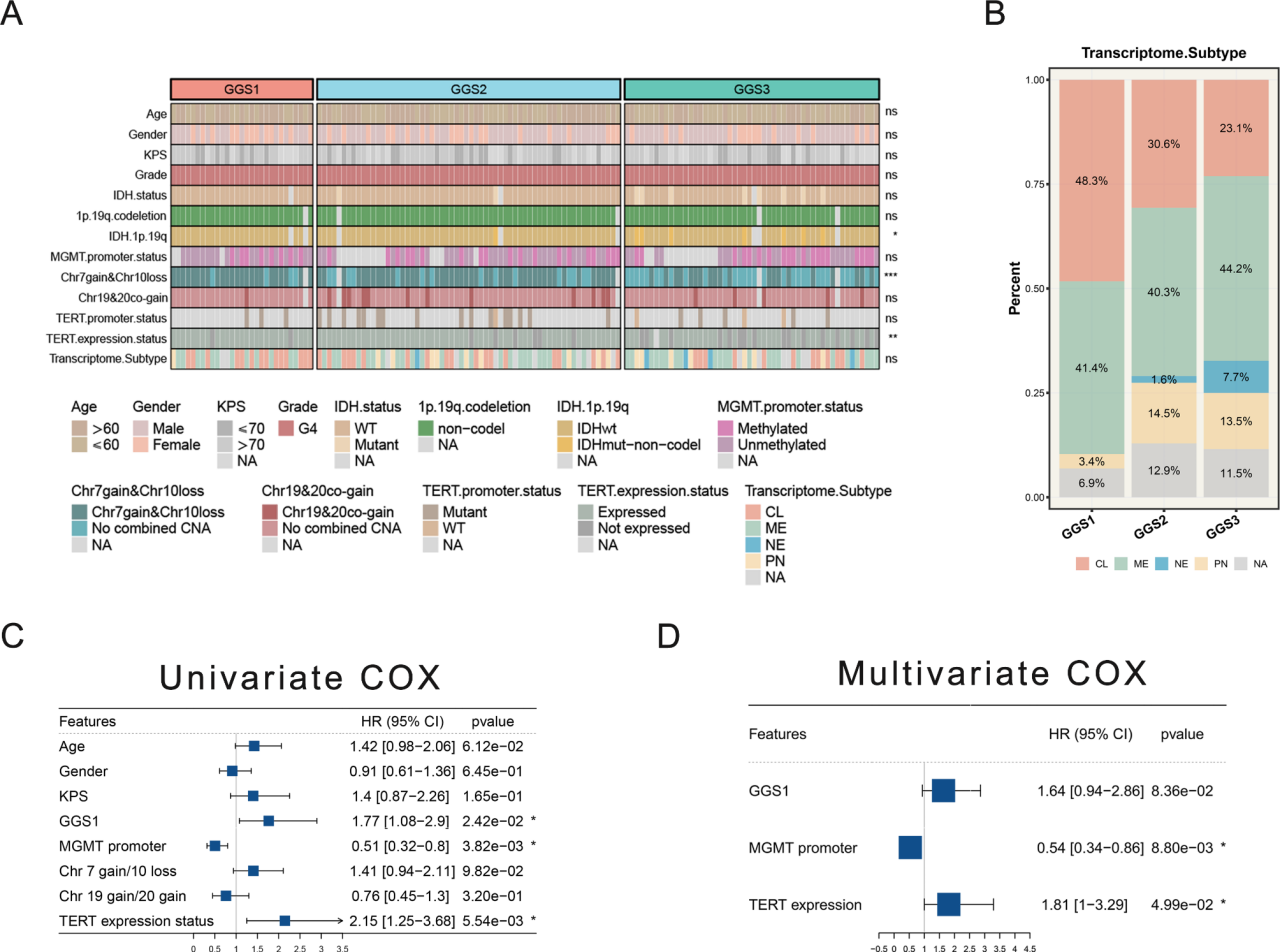
**Comparison with recognized characteristics of GBM. (A)** A heatmap was generated to display the association between GGS1 and recognized characteristics, as well as previous classification. **(B)** The distribution of previous types among GGS1-3. **(C)** Univariate analysis was performed to investigate the association of GGS1 and recognized characteristics with prognosis. **(D)** Multivariate analysis was conducted to evaluate the relationship between GGS1 and recognized characteristics with prognosis

In this study, we examined the distribution of various known features in GBM, including age, gender, Karnofsky Performance Status (KPS), grade, IDH status, 1p/19q co-deletion, chr 7 gain & chr 10 loss, chr 19 & 20 co-gain, TERT promoter status, TERT expression status, MGMT promoter methylation, and transcriptome subtypes (Fig. 5A). We found that the proportion of IDHwt-non-codel was significantly increasing and the proportion of chr 7 gain & chr 10 loss was significantly decreasing from GGS1 to GGS3. At the same time, GGS1 had the highest proportion of older patients (Figure S3G) and the lowest proportion of MGMT methylation (Figure S3H). In addition, The IDH of GGS1 was all wild type with grade 4. Compared to the previous classification of GBM, GGS1 has the highest proportion of classical subtypes and does not contain neural subtypes (Fig. 5B). We conducted both univariate and multivariate Cox regression analyses, which further confirmed the prognostic value of these features. GGS1 showed an HR > 1 in both univariate and multivariate analyses, indicating a poor prognosis. (Fig. 5C-D).

### Discovery of potentially specific drugs for GGS1

**Fig. 6:**
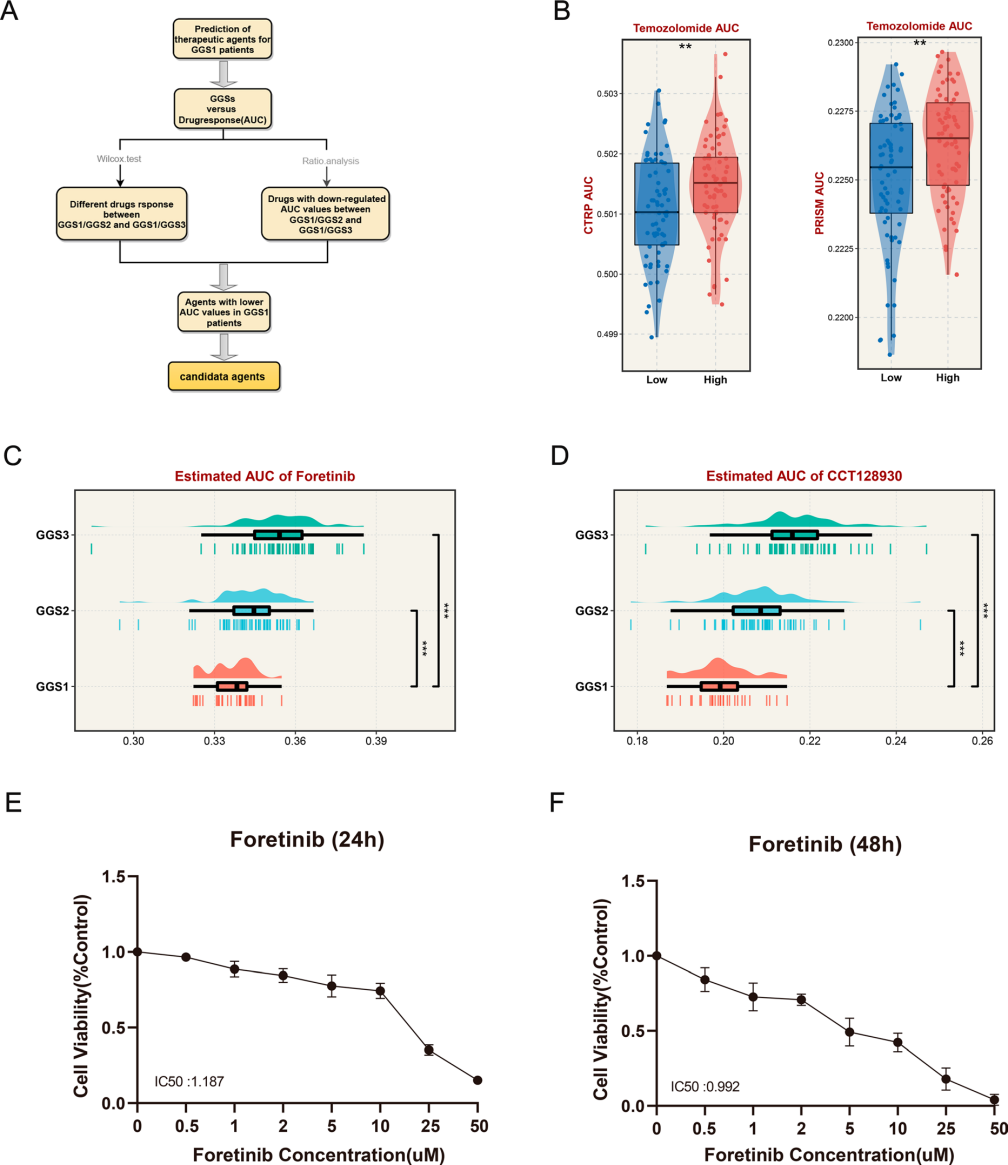
**Identification of Potential Drugs Targeting GGS1. (A)** Flow chart of drug screening. **(B)** Comparison of AUC values for Foretinib among three subtypes. **(C)** Comparison of AUC values for CCT128930 among three subtypes. **(D)** AUC comparison between high and low ADM expression groups. **(E-F)** U251 cells were seeded in 96-well plates and treated with Foretinib (0, 0.5, 1, 2, 5, 10, 25, and 50 µM) for 24 and 48 h

Using drug sensitivity data and gene expression data from PRISM and CTRP, we identified potential therapeutic agents for GGS1 (Fig. 6A). A ridge regression model was employed via the pRRophetic package to deduce the AUC value corresponding to the drug. A lower AUC value indicated more sensitivity to the drug. TMZ is the first-line drug for glioma treatment, and previous studies [26] have shown that low ADM expression improves glioma sensitivity to TMZ. Our results showed that samples with low ADM expression had lower AUC values, confirming the rationality of our method in glioma (Fig. 6B). We divided the samples into two groups according to the level of ADM expression, and the results showed that samples with low ADM expression had lower AUC values both in CTRP and PRISM, which were more sensitive to TMZ. Following the same approach, we identified potential drugs CCT128930 and Foretinib for GGS1 (Fig. 6C-D). To evaluate the sensitivity and specificity of the drug for GGS1 isoforms, we relied on publicly available cell line expression data (https://depmap.org/) and utilized the NTP algorithm to determine the corresponding cell lines for each subtype (Table S5). And, the CCK8 assay demonstrated that Foretinib exhibited a high sensitivity to U251 belonging to the GGS1 subtype, with IC50 values of 1.187 µM (24 h) and 0.992 µM (48 h), respectively (Fig. 6E-F).

## Discussion

As the most malignant tumor, GBM exhibits a high degree of intratumoral heterogeneity [2]. There is an urgent need for risk stratification and refined management of GBM. In this study, we employed Consensus Clustering, previously used in GBM, to establish three subtypes (GGS1-3) with varying prognoses based on growth-related genes. All 12 growth-related genes are poor prognostic factors and are highly expressed in GGS1, which has the worst prognosis. In contrast to adjacent non-cancerous samples within the TCGA cohort, we observed that these 12 growth-related genes were predominantly expressed at high levels in cancer tissues. In addition to this, these genes are associated with DNA replication and cell proliferation. As a component of the RPA/RP-A complex, RPA3 binds and stabilizes ssDNA intermediates that arise during DNA replication or stress, preventing reannealing. Additionally, it recruits and activates various DNA metabolism proteins and complexes, playing a vital role in both DNA replication and the cellular DNA damage response [27]. HSPA5 may play important roles in regulating apoptosis and cell proliferation [28]. Although the specific mechanism of growth-related genes in tumor development is not the focus of this paper, we have conducted an in-depth exploration of the characteristics of GGS1, particularly in copy number variations and biological function. In conclusion, our study presents the first classification of GBM based on growth-related genes and identifies a more malignant subtype: GGS1.

The stability of molecular subtypes is crucial for their generalization and clinical application. In this study, we confirmed the stability of growth-related genes subtypes (GGSs) in three GEO cohorts and an in-house cohort. We used the NTP algorithm to predict sample subtypes and validate the confidence of cohort predictions, which is a reliable method [16]. Signature genes were selected through the analysis of inter-subtype differences. The NTP algorithm was utilized to validate the prediction of cohort subtypes, based on the aforementioned signature genes. The GGSs showed stability in all GEO cohorts and the ZZ cohort, and the prognosis of the validation cohort subtypes was consistent with that of the TCGA cohort, and each subtype signature gene was also similarly expressed. This suggests that our classification is not biased by the TCGA samples’ contingency, indicating potential for clinical translation and generalizability.

Based on the 2021 WHO classification of central nervous system tumors, adult IDHwt diffuse astrocytoma can be classified as glioblastoma (GBM), even in the absence of microvascular proliferation or necrosis, if they exhibit at least one of the following molecular markers: TERT promoter mutation, EGFR gene amplification, or chr 7 gain & chr 10 loss [29]. There are several lines of evidence [30, 31] indicating that the presence of one or more of these three markers is sufficient to assign the highest WHO grade. Moreover, all of these factors are linked to poor prognosis in GBM [32, 33]. Notably, the EGFR gene is situated on chr 7 [32]. In our study, all samples of GGS1 were of the IDHwt type, and the proportion of TERT promoter mutation, EGFR gene amplification, and chr 7 gain & chr 10 loss were the highest among the three types. Although GGS2 also exhibited a higher level of chr 7 gain & chr 10 loss, the proportion of chr 19 & 20 co-gain was also higher. The presence of chr 19 & 20 co-gain is indicative of a better prognosis, which may account for the superior prognosis of GGS2 as compared to GGS1 [34]. Further investigation is necessary to establish the connection between chr 7 gain & chr 10 loss and chr 19 & 20 co-gain, as well as their underlying biological significance. In conclusion, GGS1 represents a molecular subtype that demonstrates a greater degree of similarity with GBM diagnosis in terms of copy number variations.

Growth-related genes screened by CRISPR-Cas 9 have already been used in several studies [9, 10]. GGSs were established based on growth-related genes of GBM obtained by CRISPR-Case 9 screening, and we found that growth-related genes of GBM all were indicators of poor prognosis. More interestingly, these growth-related genes all are highly expressed in GGS1. GSEA and GSVA enrichment analysis shows that GGS1 is more active in proliferative function and metabolic function. The Phosphatidylinositol 3-kinase/Akt/mammalian target of the rapamycin (PI3K/Akt/mTOR) pathway is a crucial therapeutic target in cancer therapy. This signaling pathway is essential for regulating processes such as cell growth, proliferation, and survival [35]. It is noteworthy that in 88% of GBM cases, this signaling pathway is constitutively activated, and the upstream protein responsible for this activation is EGFR [36]. Consequently, targeting this pathway has become a major focus in the effort to treat GBM [37]. Furthermore, the high expression of EGFR in GGS1, at both the transcriptome and protein levels relative to the other two types, suggests that GGS1 exhibits a higher degree of proliferation. Notably, EGFR can also influence the TERT promoter mutation region through GABPA and GABPB1, which can in turn promote proliferation and immortality [25]. In summary, at the biological level, GGS1 exhibits distinct high proliferative characteristics.

GGS1 consistently demonstrated the most unfavorable prognosis across three independent cohorts. Additionally, in both univariate and multivariate COX analysis, its hazard ratios (HRs) were consistently greater than 1, indicating a poor prognostic indicator. Faced with the current situation, our research team aimed to develop drugs that specifically target GGS1. Through the analysis of PRISM and CTRP databases, we have identified CCT128930 and Foretinib as potential drugs that show higher sensitivity to GGS1. Notably, CCT128930 is an AKT enzyme inhibitor [38] that has shown promising results in clinical trials for pediatric tumors associated with the AKT pathway [39]. Similarly, Foretinib has demonstrated efficacy in inhibiting medulloblastoma [40] and has been shown to have an inhibitory effect on GBM growth by inhibiting the G2/M cell cycle [41]. More importantly, Foretinib showed high sensitivity to U251 (GGS1 subtype), with IC50 values of 1.187 µM (24 h) and 0.992 µM (48 h), respectively. These values indicate that Foretinib exhibits strong inhibitory activity that surpasses the previously reported inhibition of U251 by LTr1 (2.03µM, 24 h) [42]. These findings emphasize the superior therapeutic potential of Foretinib.

We conducted genome-scale CRISPR-Cas9 screening and univariate COX analysis to identify growth-related genes for risk stratification. This approach aligns with the concept of precise patient management in clinical practice and can guide decision-making and improve clinical treatment efficacy to some extent. Therefore, our findings hold a certain value. However, this study has certain limitations. The mechanism by which growth-related genes contribute to a more malignant phenotype in GBM requires further investigation. Additionally, we used bioinformatics methods to predict the chemosensitivity of GGS1, which necessitates long-term follow-up in prospective clinical studies for further validation. More critically, as research progresses, it has become evident that the prognosis of glioma patients can be influenced by factors such as primary and recurrent tumor status, chemoradiotherapy, and IDH status [29]. However, due to temporal limitations, certain datasets may not contain the relevant information pertaining to these factors. If conditions permit, to better investigate GBM, we intend to base primary IDHwt GBM on the next study while trying to maintain consistency of treatment measures such as chemoradiotherapy in an attempt to reduce the impact of sample bias on the results.

## Conclusion

We have identified three prognostic subtypes based on growth-related genes, and have observed differences in copy number variations and biological functions among them. Furthermore, we conducted investigations into the underlying mechanisms contributing to the unfavorable prognosis of GGS1 and identified potentially responsive therapeutic agents. These findings represent a novel contribution to the field, as previous studies have not reported similar results. Our discoveries deepen our understanding of copy number variations and biological function in GBM, clarify its heterogeneity, and offer insights into precision treatment.

### Electronic supplementary material

Below is the link to the electronic supplementary material.

Supplementary Material 1

Supplementary Material 2

Supplementary Material 3

Supplementary Material 4

## Data Availability

Public data used in this work can be acquired from the TCGA (https://portal.gdc.cancer.gov/), GEO (https://www.ncbi.nlm.nih.gov/geo/), Cell line expression data (https://depmap.org/portal/download/all/), and TCPA (https://www.tcpaportal.org/tcpa/), Other data supporting the findings of this study are available from the corresponding author upon reasonable request.

## Abbreviations

GGSs: Growth-related genes subtypes
GBM: Glioblastoma
NTP: Nearest template prediction
AUC: Area under the dose-response curve
DepMap: Dependency Map
GISTIC2.0: Genomic identification of significant targets in cancer 2.0
PRISM: Profiling of relative inhibition simultaneously in mixtures
CTRP: Cancer therapeutics response portal
