# Supplementary figures and images for: CRISPR-Cas9 identifies growth-related subtypes of glioblastoma with therapeutical significance through cell line knockdown
Source: BMC Cancer. 2023 Aug 14;23:749. doi: 10.1186/s12885-023-11131-7 (PMC10424363; doi:10.1186/s12885-023-11131-7)

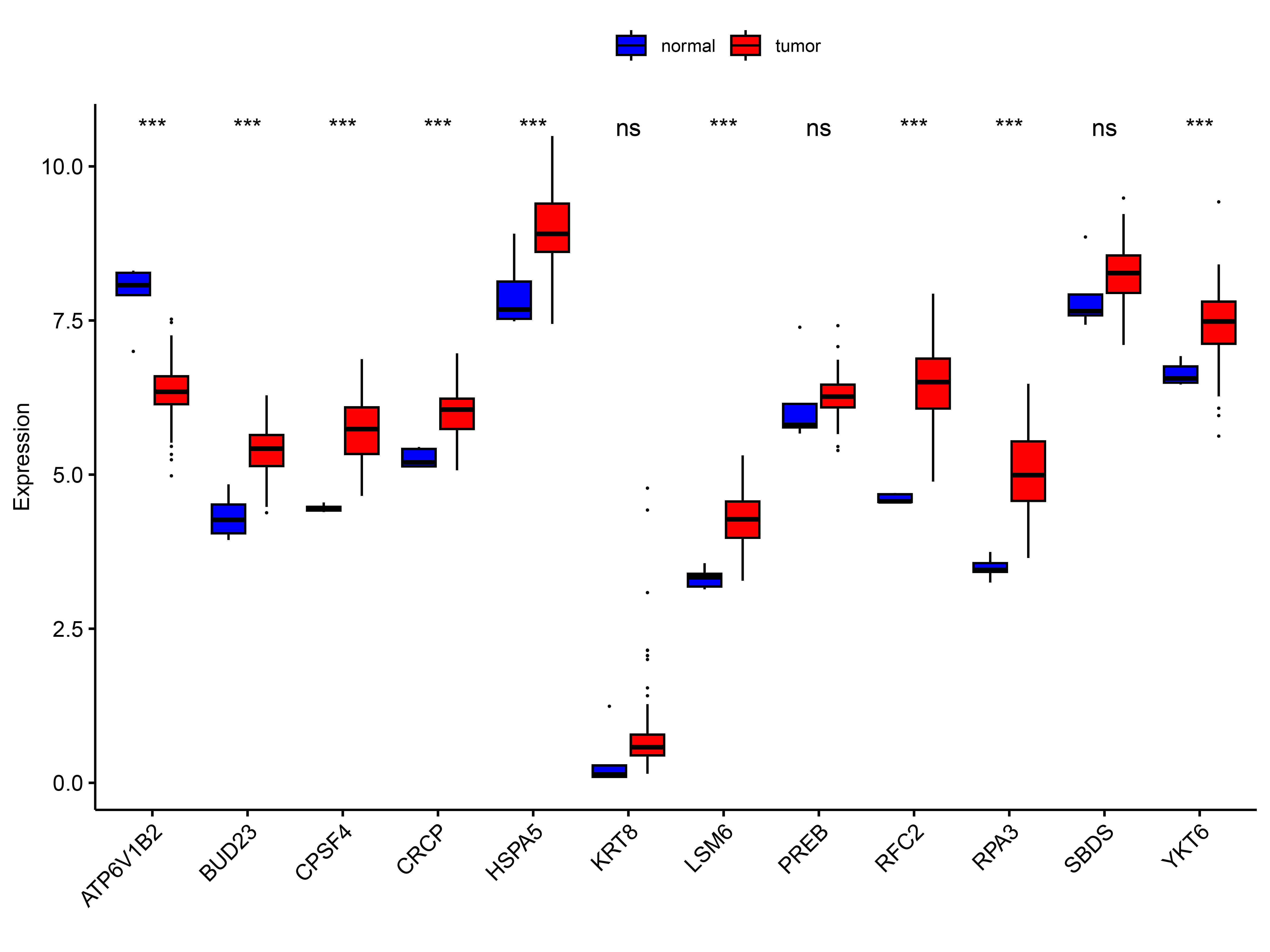

Supplement: Supplementary file 1 — Supplementary Material 1 [file 12885_2023_11131_MOESM1_ESM.png]

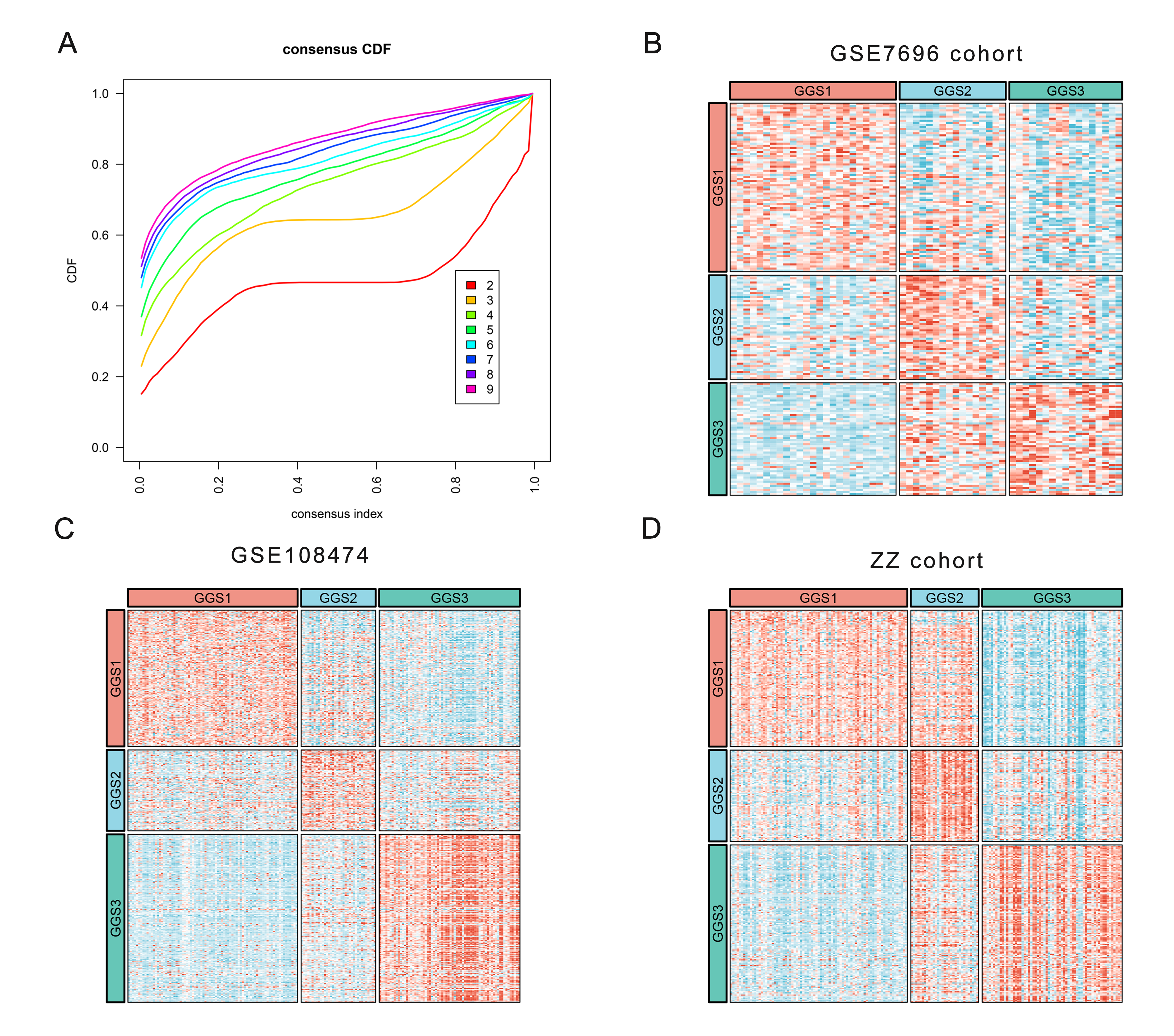

Supplement: Supplementary file 2 — Supplementary Material 2 [file 12885_2023_11131_MOESM2_ESM.png]

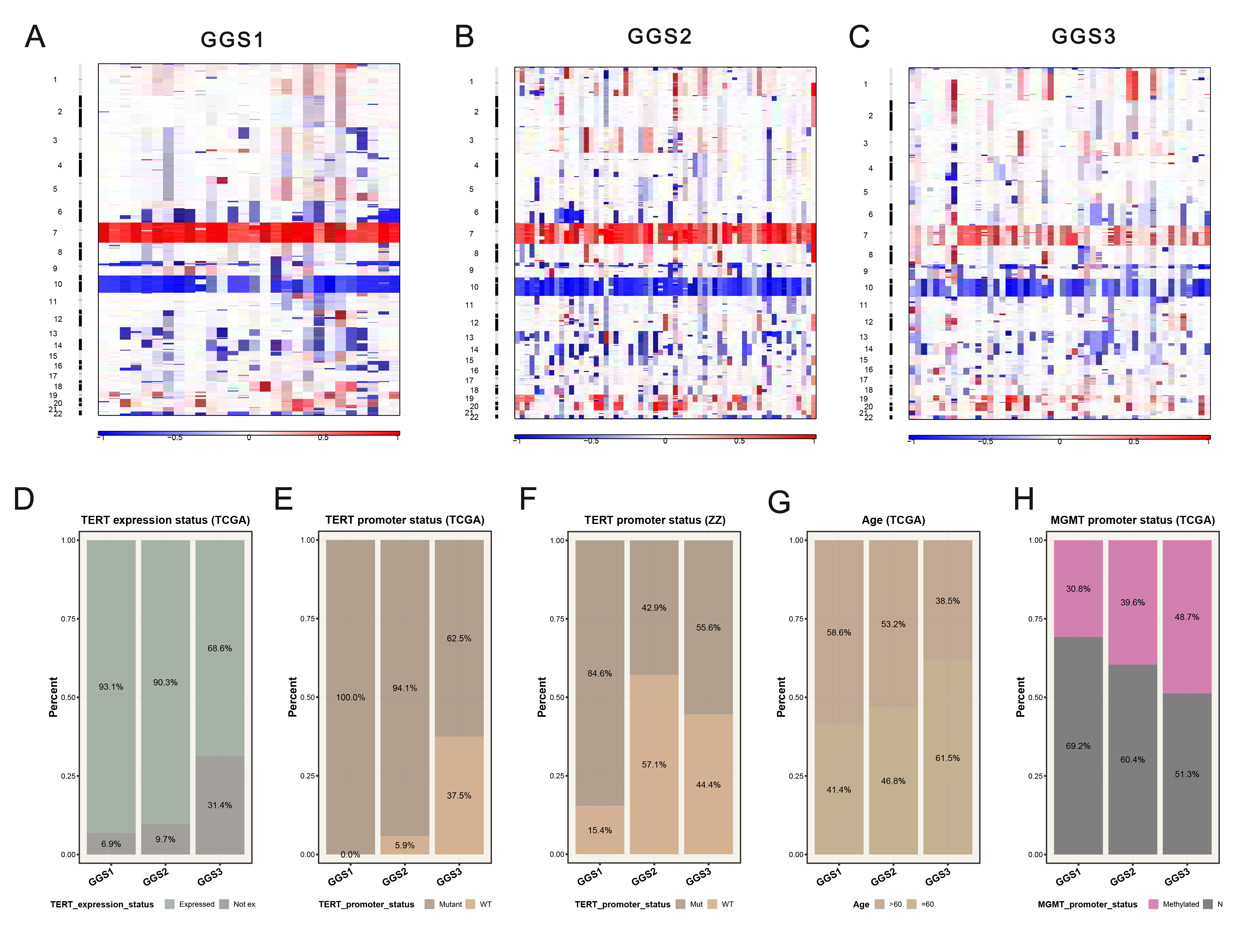

Supplement: Supplementary file 3 — Supplementary Material 3 [file 12885_2023_11131_MOESM3_ESM.png]
